# Analysis of Insect Resistance and Ploidy in Hybrid Progeny of Transgenic *BtCry1Ac* Triploid Poplar 741

**DOI:** 10.3390/plants14162563

**Published:** 2025-08-18

**Authors:** Yan Zhou, Hongyu Cai, Renjie Zhao, Chunyu Wang, Jun Zhang, Minsheng Yang, Jinmao Wang

**Affiliations:** 1 Forest Tree Genetics and Breeding Laboratory, College of Forestry, Hebei Agricultural University, Baoding 071001, China; zhouyan202506@163.com (Y.Z.); caihy0817@163.com (H.C.); renjie612@163.com (R.Z.); chunyuwang2024@163.com (C.W.); zhangjunem@126.com (J.Z.); 2 Faculty of Life Sciences, Northeast Agricultural University, Harbin 150030, China

**Keywords:** transgenic *poplar*, *BtCry1Ac* gene, sexual hybridization, F1 interspecific, aneuploid

## Abstract

With the increasing severity of forest pest problems, breeding insect-resistant varieties has become a crucial task for the sustainable development of forestry. The highly insect-resistant triploid Populus line Pb29, genetically modified with *BtCry1Ac*, served as the maternal parent in controlled hybridization with three paternal Populus cultivars. Hybrid progenies were obtained through embryo rescue and tissue culture. Results showed that 4 °C storage was favorable for pollen preservation, with 84K poplar exhibiting superior pollen viability and embryo germination rates. All progenies displayed significantly lower seedling height and ground diameter growth than the maternal parent (*p* < 0.05), with some showing leaf shape and branching variations. Among the three crosses, the 84K-sired progeny exhibited the best growth performance but the highest variability. PCR analysis confirmed stable inheritance of the *BtCry1Ac* and *Kan* genes from Pb29, showing tight linkage. Progenies carrying *BtCry1Ac* exhibited detectable gene transcription and toxic protein accumulation, though expression levels varied due to copy number, insertion sites, and potential co-suppression effects. Ploidy analysis suggested all hybrids were aneuploid, with lower survival rates than the maternal parent. Insect-feeding assays confirmed high resistance in all *BtCry1Ac*-inheriting progenies, with an average larval mortality rate of 97.03%. Mortality rates and death indices significantly correlated with transcript abundance and toxin protein levels. These results demonstrate that *BtCry1Ac* insect resistance is stably inherited through hybridization. Transgene expression appears co-modulated by copy number, insertion sites, and ploidy status. Simultaneously, it was found that the aneuploid progeny derived from triploid hybridization exhibited growth disadvantages. This provides an important basis for subsequent poplar improvement breeding.

## 1. Introduction

Insect pests of agricultural and forestry crops pose a persistent threat to agricultural production. Among them, transgenic technology, as a precise genetic engineering tool, has become a key strategy for crop trait improvement and is widely applied in the field of insect-resistant breeding [1]. The strategy of utilizing insecticidal toxin genes derived from *Bacillus thuringiensis* (*Bt*) in transgenic crops to control major pests has a practical history of over twenty years [2]. In the field of insect-resistant poplar research, China has made breakthrough progress in the development and application of transgenic *Bt* insecticidal toxin poplars. In 2000, Ying-chuan T et al. transformed a modified *BtCry1Ac* gene into poplar 741 [*P. alba* L. × (*P. davidiana Dode. + P. simonii Carr.*) *× P. tomentosa Carr*] and successfully obtained highly resistant transgenic plants [3]. Since 2002, transgenic *Bt* white poplars have been commercially cultivated in China, and field trials have demonstrated stable insect resistance [4]. Based on the established foundation of transgenic *Bt* poplar research, scientists have conducted multi-gene transformation studies to obtain new poplar germplasm with multiple resistance traits and systematically carried out comprehensive research, including biosafety assessment and detection of the unintended effects of transgenesis. Huo Xuemei [5] introduced the *Cry3A* gene into the transgenic poplar 741 line (Pb29) obtained by Tian Yingchuan via secondary transformation, creating a new transgenic poplar 741 line simultaneously containing *Cry3A*, *Cry1Ac*, and *API* genes. Wang Guiying et al. conducted systematic insect resistance evaluations of these transgenic lines, confirming that the double *Bt* gene-transformed lines could not only simultaneously detect the expression of *Cry1Ac* and *Cry3A* toxin proteins but also exhibited high lethality against both the lepidopteran *Hyphantria cunea* and the coleopteran *Plagiodera versicolora*, achieving an expansion of the insect resistance spectrum and a gene stacking effect [6]. Ren et al. simultaneously transformed the *Cry1Ac* and *Cry3A* genes into *Populus × euramericana* ‘Neva’ and investigated the expression of foreign genes and insect resistance [7]. Wang et al. analyzed differences in arthropod communities, target arthropods, and non-target arthropods between transgenic *BtCryIAc* and non-transgenic experimental stands of *Populus × euramericana cv.* ‘74/76’, finding that transgenic poplars had no significant adverse effects on non-target arthropods [8]. Wu et al. found that the integration of foreign genes led to differences in the response to drought stress between transgenic double *Bt* (*Cry1Ac*, *Cry3A*) *Populus × euramericana* and wild-type (WT), with transgenic poplars exhibiting poorer drought resistance [9].

Hybrid breeding is an important breeding method for poplars, and a large number of new poplar hybrid varieties have been developed and widely used in production. Combining transgenic breeding with hybrid breeding is a worthwhile direction to explore for poplar breeding. To create new poplar germplasm combining high-efficiency insect resistance and ecological adaptability, we can conduct sexual hybridization between transgenic varieties that have completed safety assessments and commonly used elite poplar varieties, creating superior germplasm, inheriting foreign genes for the selection and breeding of excellent poplar varieties. Current research has explored hybridization between transgenic crops and wild-type varieties: Shao et al. reported transgene silencing in backcross progenies between transgenic oilseed rape (*Brassica napus*) crops and wild relatives [10]; Wang et al. found that transgenes in oilseed rape can be stably inherited and expressed through hybridization and backcross generations and survive in nature [11]; Jiang et al. found that advanced crop–wild rice (*Oryza rufipogon*) hybrids overexpressing the *EPSPS* transgene exhibited significantly enhanced soil seed bank longevity and dormancy characteristics [12]; Zhang et al. confirmed that the growth and fecundity of hybrid progenies between transgenic soybean and wild soybean were significantly affected [13]. Although the aforementioned studies generally adopt a cautious stance towards gene flow from transgenic crops, this phenomenon also provides a potential opportunity for creating novel germplasm resources for agriculture and forestry. Due to aneuploid chromosome numbers, triploid plants exhibit high irregularity during meiosis, producing gametes with varying chromosome numbers, which can lead to the formation of polyploid and aneuploid plants in their progeny [14,15]. The novel phenotypes arising from aneuploidy hold significant value for germplasm innovation. Particularly in plants with narrow genetic backgrounds, utilizing heteroploids can rapidly increase genetic diversity and cultivate new varieties [16]. Yasuda et al. found that leaf, flower and fruit traits in aneuploid citrus progeny and true triploid intergeneric hybrids after hybridization were mostly intermediate, but fruit size and flowering habits were closer to the genetic characteristics of *Fortunella crassifolia Swingle* [15]. Singh et al. analyzed the role of gliadin in the genetic susceptibility to celiac disease using aneuploid lines of tetraploid wheat (*Triticum turgidum var durum*, AABB) [17]. Jin et al. obtained haploid homozygous diploids and aneuploids of sour orange (*Citrus aurantium* L.) through anther culture, providing a foundation for citrus variety improvement [18]. The above studies indicate that aneuploid variation associated with phenotypic traits has significant potential in crop variety development, genetic research, and chromosome engineering [19]. This study used the highly resistant transgenic *BtCry1Ac* triploid poplar 741 line Pb29 as the maternal parent and conducted artificial hybridization with *Populus* 84K, *Populus leucopyramidalis*, and *Populus × euramericana* Male No. 1 as paternal parents. By screening the hybrid progeny, new insect-resistant poplar germplasm with different ploidy levels was rapidly obtained, aiming to provide a theoretical basis for insect-resistant poplar breeding and the cultivation of aneuploid varieties.

## 2. Results

### 2.1. Comparative Analysis of Pollen Storage Temperatures and Paternal Line Viability

**Figure 1:**
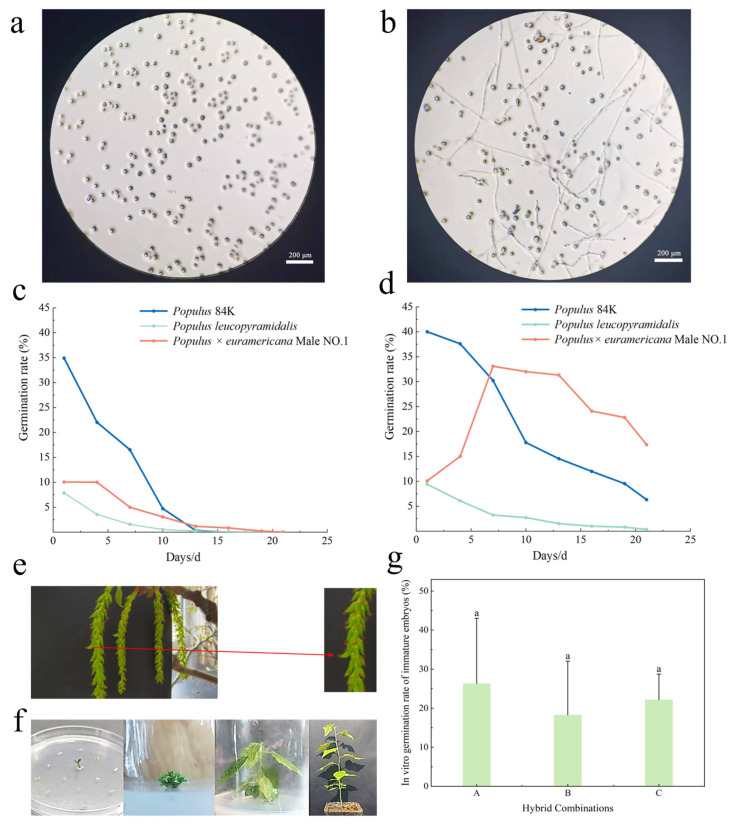
Comparative analysis of pollen germination dynamics and post-pollination embryonic development in hybrid poplar lines. Note: figure (**a**) shows the pollen traits in the non-germinating state, and figure (**b**) shows the pollen traits in the germinating state; figure (**c**) shows the pollen germination rates of different poplar varieties under room temperature storage (25 °C), and figure (**d**) shows the pollen germination rates of different poplar varieties under refrigerated storage (4 °C); figure (**e**) shows the embryonic development after pollination, the red line indicates the capsule with locally magnified embryonic development and (**f**) shows the growth process after embryo germination. figure (**g**) shows the germination rate of immature embryos in the progeny of different hybrid combinations, and A, B, and C are the hybrid combinations: A is Pb29 × 84K, B is Pb29 × *Populus × euramericana* Male NO. 1, and C is Pb29 × *Populus leucopyramidalis*, the same below. Different letters indicate significant differences between treatment groups (*p* < 0.05, Fisher’s LSD test), the same below.

Pollen preservation and viability are crucial for hybrid breeding [20]. Microscopic observation showed (Figure 1a,b) that poplar pollen grains have a relatively small particle size and can germinate to form slender pollen tubes under suitable conditions. A comparison of pollen viability changes in the three paternal materials at different temperatures showed (Figure 1c) that under 25 °C storage, 84K poplar had the highest initial germination rate at 34.91%, significantly higher than *P. × euramericana* Male No. 1 (10.05%) and *Populus leucopyramidalis* (7.87%). Pollen viability decayed rapidly under room temperature storage, with pollen from all three paternal parents completely losing viability within 16–21 days. In contrast, low-temperature storage at 4 °C significantly prolonged pollen longevity (Figure 1d). After 21 days of storage, *P. × euramericana* Male No. 1 still maintained a germination rate of 17.36%, while 84K poplar was at 6.33%. Unlike the declining trend in the germination rate seen in the other two paternal parents, the pollen viability of *P. × euramericana* Male No. 1 under low temperature showed an initial increase followed by a decrease, peaking at a germination rate of 33.09% on day 10. Comprehensive comparison showed that 84K poplar had the best pollen viability characteristics. This study confirmed that storage at 4 °C is an effective method for maintaining poplar pollen viability.

### 2.2. Artificial Pollination, Hybridization, and Hybrid Embryo Rescue

Poplar 741 is a triploid variety, highly sterile under natural conditions, making it difficult to obtain its mature seeds. Immature embryo culture is an effective way to obtain hybrids. Ten days after pollination, the development of zygotic embryos within the capsules was observed. As shown in Figure 1e, the capsule apex of seed-developed fruits curves upward, whereas capsules without developing embryos lack this characteristic. At 14–21 days after pollination, immature hybrid progeny embryos were excised. The excised embryos were inoculated onto Petri dishes containing medium, with an average of 20 embryos per dish (as shown in Figure 1f). Different hybrid combination progeny embryo seedlings were obtained through tissue culture. Through artificial hybridization and embryo rescue, totals of different numbers of germinated immature embryos were obtained from the three hybrid combinations: 60 for combination A, 25 for B, and 19 for C. However, a large number of immature embryos died during the tissue culture process. The final number of successfully obtained tissue-cultured seedling clones was 17 for combination A (Pb29 × 84K), 10 for B (Pb29 × *Populus × euramericana* Male NO. 1), and 7 for C (Pb29 × *Populus leucopyramidalis*). After transplantation to the field, the number of surviving progeny clones for each hybrid combination further decreased: eight clones survived for combination A, and six clones each survived for combinations B and C. An analysis of the in vitro culture of immature embryos from each hybrid combination revealed (Figure 1g) that the cross-compatibility between different paternal parents and Pb29 differed, but the difference was not statistically significant (*p* > 0.05, the same below): The progeny immature embryo germination rate was highest for the 84K combination (26.33%), followed by *Populus leucopyramidalis* (22.16%), and lowest for *P. × euramericana* Male No. 1 (18.26%). This result indicates varying degrees of embryo abortion in hybrid combinations using transgenic poplar 741 as the maternal parent. The underlying cause may be the unequal chromosome segregation during meiosis in the triploid, leading to high sterility. Additionally, subtle differences were observed in the in vitro culture efficiency of immature embryos among different hybrid combinations.

### 2.3. Growth Analysis of Progeny from Different Hybrid Combinations

**Figure 2:**
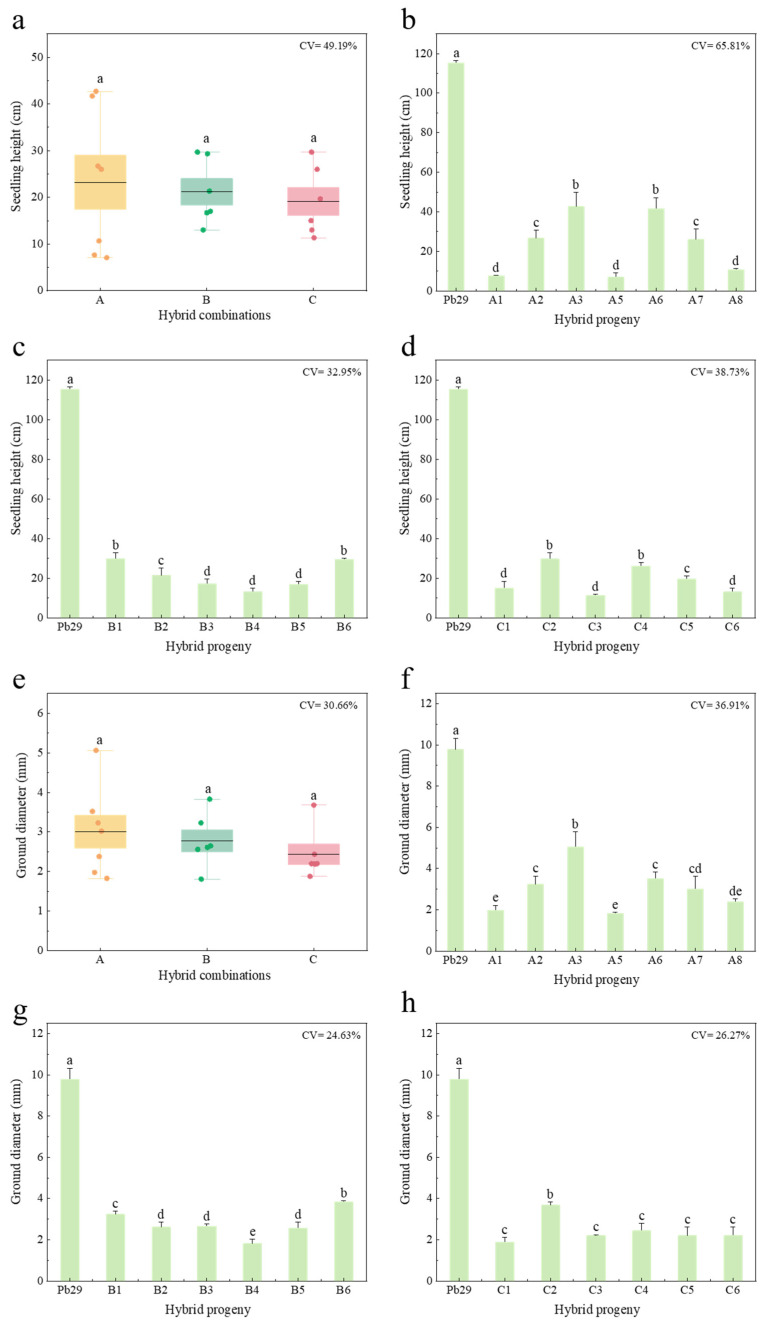
Comparison of seedling height and ground diameter of different hybrid combination progenies. Note: figure (**a**) shows the comparison of seedling height among different hybrid combinations, while (**b**–**d**) present the intra-combination seedling height comparisons for A, B, and C progenies, respectively. figure (**e**) illustrates the comparison of ground diameter among different hybrid combinations, and (**f**–**h**) show the intra-combination ground diameter comparisons for A, B, and C progenies, respectively; A4 died in the field.

The measurement results of the seedling height and ground diameter of hybrid progenies two months after successful planting showed (Figure 2) that the seedling height and ground diameter of all hybrid progenies were significantly lower than those of the maternal parent Pb29 (height, 115.33 cm; ground diameter, 9.78 mm). Although the growth traits of progeny among different hybrid combinations did not reach a significant difference level, obvious growth trend differences were observed. Progeny with 84K as the paternal parent (A) performed best, with an average seedling height of 23.98 cm and an average ground diameter of 3.05 mm. *P. × euramericana* Male No. 1 (B) was next, with a progeny average height of 21.73 cm and a ground diameter of 2.84 mm. Progeny with *Populus leucopyramidalis* as the paternal parent (C) showed relatively lagging growth performance, with an average height of 19.11 cm and a ground diameter of 2.25 mm. Significant variation existed among individual progenies within each combination. For combination A, the coefficient of variation (CV) for height (65.81%) and ground diameter (36.91%) was higher than the inter-combination CV for height (49.19%) and ground diameter (30.66%). Growth indicator differences among individuals in group A were particularly prominent. Seedling height measurement data showed that the tallest individual A3 (42.67 cm) differed by 6.03 times from the shortest individual A5 (7.07 cm). Ground diameter measurements showed a similar trend; individual A3 had the largest ground diameter (5.06 mm), which was 2.76 times higher than the minimum value (A5, 1.83 mm). The CVs for height and ground diameter in combinations B and C were lower than the inter-combination variation level. For combination B, the height and ground diameter CVs were 32.95% and 24.63%, respectively; for C, they were 38.73% and 26.27%, respectively. These results indicate that the paternal genotype may influence the growth traits of hybrid progeny through genetic interaction effects. When 84K poplar was used as the paternal parent, it exhibited a strong growth-promoting effect, and its progeny population had richer phenotypic variation.

A comparative analysis of leaf traits between hybrid progeny and Pb29 seedlings revealed significant differences in phenotypic expression among the progeny clones. The leaf growth phenotypic expression of hybrid progeny was significantly inferior to Pb29. Many progenies exhibited leaf margin curling, increased branching, and stem bending.

**Figure 3:**
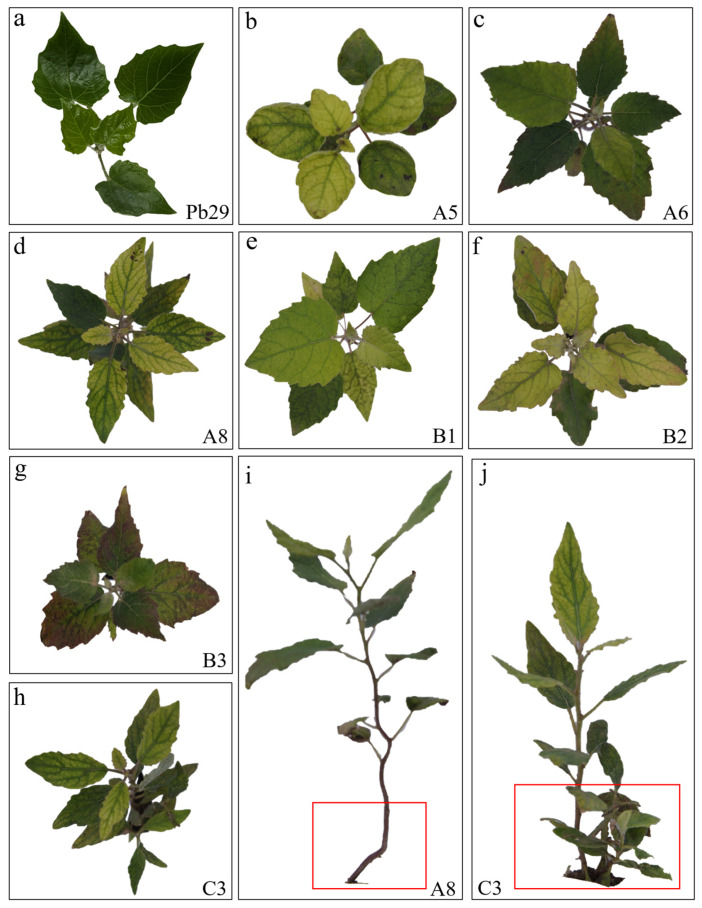
Morphological characteristics of some progeny clones. Note: figure (**a**) shows the the Pb29 clone, figure (**b**) shows the A5 clone, figure (**c**) shows the A6 clone, figure (**d**) shows the A8 clone, figure (**e**) shows the B1 clone, figure (**f**) shows the B2 clone, figure (**g**) shows the B3 clone, figure (**h**) shows the C3 clone, the red box in figure (**i**) shows the stem bending pattern of A8 clone, and the red box in the figure (**j**) shows the basal lateral branches of the C3 clone.

Observation revealed that hybrid progeny clones exhibited significant leaf morphological variation compared to the maternal parent Pb29 (Figure 3a). The maternal parent Pb29 had leaves with a typical triangular to ovate–triangular shape, characterized by a truncate or shallowly cordate base, an acuminate apex, and a serrated margin. In contrast, the progeny displayed rich phenotypic diversity: A5 (Figure 3b) had ovate leaves with inwardly and smoothly curled margins; A6 (Figure 3c) exhibited abnormal morphology including bending of the lower stem and elongated internodes. Its lanceolate leaves had significantly higher serration density than the maternal parent, and the leaf apex showed unique features of being sharply pointed upwards or drooping; A8 (Figure 3d) was characterized by a compact plant type with small lanceolate leaves and shortened internodes, and the stem near the ground was curved (Figure 3i). Among group B progenies, B1 (Figure 3e) had thick leaves with a sharp apex and cordate base; B2 (Figure 3f) exhibited deeply undulate leaf margins accompanied by obvious differences in color between the upper and lower leaf parts; B3 (Figure 3g) showed compact ovate leaves with reddish margins. C3 not only formed a unique combination of slender lanceolate leaves and an obtuse-rounded leaf base but also exhibited the special phenotype of basal lateral branches (Figure 3j). These hybrid progenies showed significant diversity in leaf shape (ovate and lanceolate), leaf margin characteristics (serration density and undulation degree), leaf texture (thickness), and overall plant type (internode length and branching characteristics). The widespread phenotypic variation in hybrid progeny not only reflects the recombination effect of parental genetic material but may also be related to alterations in developmental regulation caused by transgene insertion. This provides valuable material for studying the genetic regulatory mechanisms of leaf morphogenesis in poplar. In particular, the coordinated stem–leaf variation in A8 and the basal lateral branching phenomenon in C3 suggest possible changes in shoot apical meristem activity, warranting further investigation into their molecular mechanisms.

### 2.4. Detection of Foreign Genes and Insertion Sites in Hybrid Progeny

**Figure 4:**
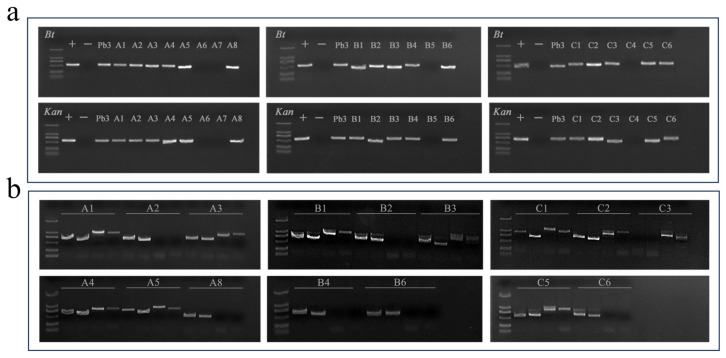
PCR analysis results for the detection of *BtCry1Ac*, *Kan* gene, T-DNA insertion site, and insertion orientation in hybrid progeny plants. Note: figure (**a**) shows the PCR detection results of the *Bt* gene and *Kan* gene for each progeny strain, A–C, in order: positive control, negative control, Pb29, and progeny strains; figure (**b**) displays the detection results of the T-DNA insertion site orientation in the progeny.

Specific primers for the *BtCry1Ac* gene and the *Kan* gene were used for PCR detection of 20 clones derived from the three hybrid combinations. A plasmid containing the target gene was used as the positive control, and wild-type poplar 741 was used as the negative control. An analysis of PCR amplification products by 1% agarose gel electrophoresis (Figure 4a) showed that among the 20 tested clones, 16 clones exhibited specific bands at 546 bp, confirming that these clones inherited the *BtCry1Ac* foreign gene from the maternal parent. PCR results showed that all progeny clones containing the *BtCry1Ac* gene also tested positive for the presence of the *Kan* gene. This result indicates that the two foreign genes show a stable linked inheritance relationship in the hybrid progeny, with no gene loss or linkage disruption occurring during generational transmission, thus confirming the genetic stability of the foreign genes in the hybrid progeny.

Based on the whole-genome resequencing and insertion site analysis of Pb29 using high-throughput sequencing (NGS) technology previously reported by Chen et al. [21], the validated specific primers from the literature were directly used for PCR amplification. This successfully verified the T-DNA insertion sites and their orientation in the hybrid progeny. Through electrophoresis detection of PCR products, the inheritance status of the two T-DNA insertion sites from the maternal parent Pb29 in each hybrid progeny was systematically identified. This provided direct molecular evidence for analyzing the inheritance pattern of foreign genes in hybrid progenies. PCR results of hybrid progenies revealed (Figure 4b) that A1, A3, A4, A5, B1, B3, C1, C2, and C5 carried both insertion sites simultaneously; A2, A8, B2, B4, B6, and C6 only detected the insertion site on chromosome 3, indicating that these progenies inherited chromosome 3 from Pb29; whereas progeny C3 specifically inherited the insertion site on chromosome 10.

### 2.5. Real-Time Fluorescence Quantitative PCR (qPCR) Detection of Progeny Carrying the BtCry1Ac Gene

An analysis of the transcription level of the *BtCry1Ac* gene by real-time fluorescence quantitative PCR (qPCR) showed that specific fluorescent signals were detectable in all progeny clones carrying this foreign gene, confirming that the *BtCry1Ac* gene can be stably inherited through hybridization and expressed normally in the progeny.

**Figure 5:**
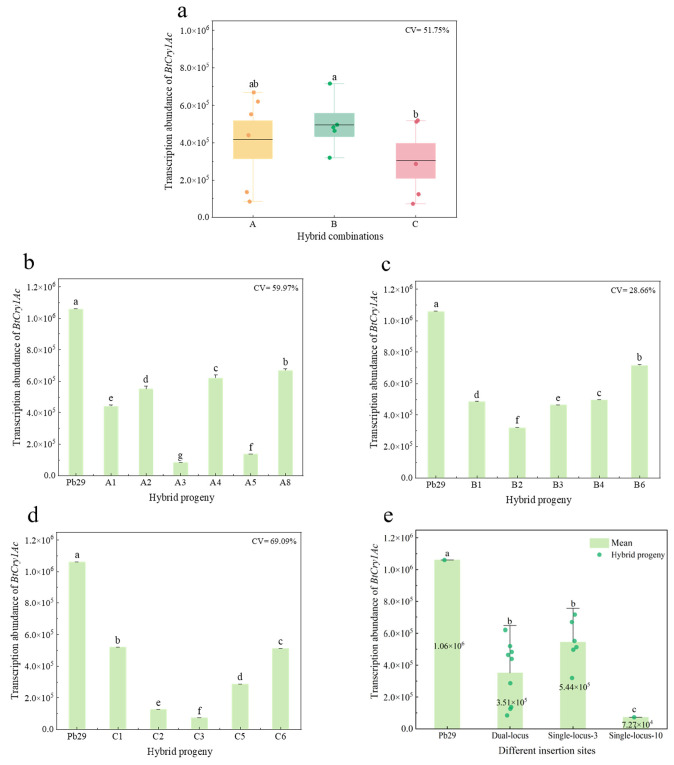
Differences in *BtCry1Ac* transcription abundance among different hybrid progeny combinations and progenies with different insertion sites. Note: figure (**a**) shows the transcription abundance of various hybrid progeny combinations; figure (**b**–**d**) depict the transcription abundance in different hybrid progeny combinations; figure (**e**) illustrates the transcription abundance of Pb29 and hybrid progeny where it is inherited to dual sites, a single site on chromosome 3, and a single site on chromosome 10.

**Figure 6:**
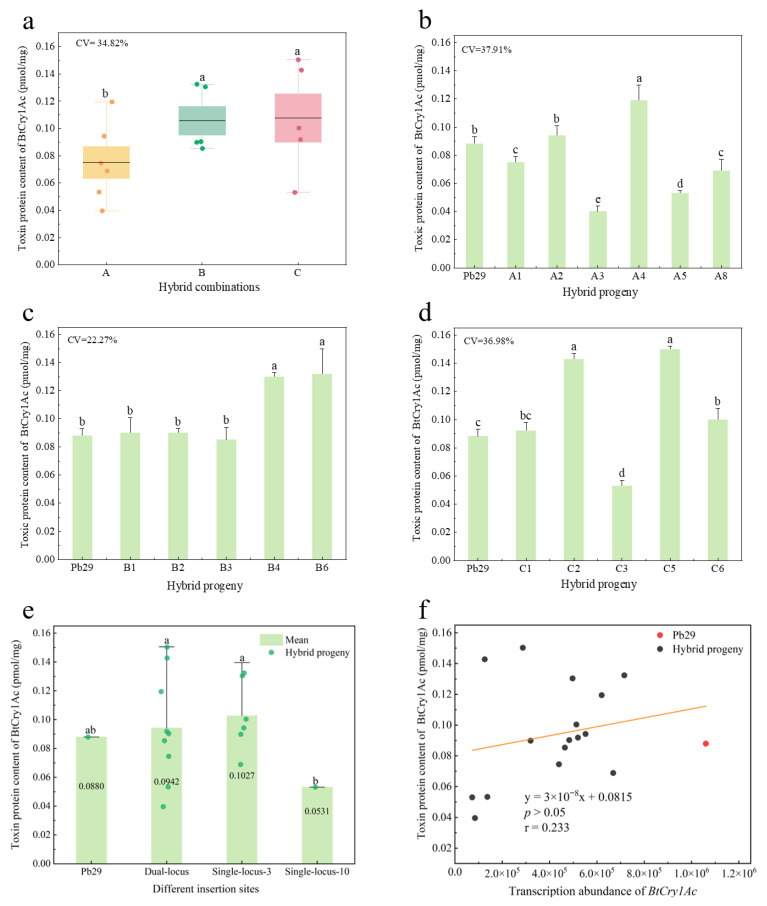
Comparison of Bt toxin protein content among different hybrid progeny combinations and at different insertion sites, along with correlation analysis between transcript abundance and toxin protein content. Note: figure (**a**) shows the toxin protein content of various hybrid progeny combinations; figure (**b**–**d**) depict the toxin protein content of different hybrid progeny combinations; figure (**e**) compares the toxin protein content of hybrid progeny with different insertion sites; figure (**f**) presents the correlation analysis between transcript abundance and toxin protein content. Regression coefficients were marked with asterisks to indicate statistical significance: * *p* < 0.05, ** *p* < 0.01, *** *p* < 0.001, unmarked coefficients were not statistically significant, the same below.

Quantitative analysis showed (Figure 5) that the maternal parent Pb29 had the highest *BtCry1Ac* gene transcript abundance (1.06 × 10^6^ copies), while the expression levels in all hybrid progenies were significantly lower than the maternal parent. Figure 6a shows that the group B hybrid progeny (with *P. × euramericana* Male No. 1 as the paternal parent) exhibited relatively higher transcript abundance (4.95 × 10^5^ copies), significantly higher than combinations A and C; group C (with *Populus leucopyramidalis* as the paternal parent) had the lowest transcript abundance (3.03 × 10^5^ copies), and group A (with 84K as the paternal parent) had an intermediate transcription level (4.17 × 10^5^ copies). Significant expression variation was observed within each hybrid combination. Group A had a coefficient of variation (CV) of 59.95% (Figure 5b), higher than the overall inter-combination variation level (51.75%). Expression differences among individuals within this group were particularly significant, with the highest-expressing individual A8 (6.69 × 10^5^ copies) differing by 7.9 times from the lowest-expressing individual A3 (8.43 × 10^4^ copies); Group B had the lowest CV (28.66%), with relatively concentrated transcript abundance among progenies within the group. The difference between the highest-expressing individual B6 (7.15 × 10^5^ copies) and the lowest-expressing individual B3 (4.63 × 10^5^ copies) was only 1.5 times (Figure 5c); Group C exhibited the most pronounced expression dispersion (CV = 69.09%). In addition to the 7.2-fold difference between the highest-expressing individual C1 (5.20 × 10^5^ copies) and the lowest-expressing individual C3 (7.27 × 10^4^ copies), the transcript abundance of other individuals also showed an obvious discrete distribution (Figure 5d). Although the expression differences among different hybrid combinations did not reach statistical significance, the experimental results preliminarily revealed a trend of the paternal genotype influencing the regulation of foreign gene expression. The specific mechanism of action requires elucidation through further molecular biology research.

Combined with foreign gene insertion site analysis (Figure 5e), the transcript abundance of the *BtCry1Ac* gene in all hybrid progeny clones (including dual-site and single-site inheritance types) was significantly lower than that of the maternal parent Pb29. The average transcript abundance for progenies inheriting dual insertion sites was 3.51 × 10^5^ copies, with an intra-group CV (56.15%) higher than the overall inter-combination variation level. The transcription level of the lowest-expressing individual A3 was similar to that of individual C3, which had a single site on chromosome 10. In contrast, progeny carrying only the chromosome 3 insertion site exhibited relatively stable high expression characteristics (average 5.44 × 10^5^ copies, CV = 25.82%). Although the average expression level of progeny with a single site on chromosome 3 was 54.99% higher than that of progeny with dual sites, and statistical testing indicated that this difference was not significant. The individual C3 with a single site on chromosome 10 exhibited extremely low transcriptional activity. Its expression level was not only reduced by 93.14% compared to the maternal parent but also reduced by 79.29% and 86.64% compared to the dual-site progeny and single-site (chromosome 3) progeny, respectively, and these differences were all statistically significant.

### 2.6. Detection of Toxin Protein Content in Progeny Carrying the Bt Gene

Quantitative detection of total protein content and the BtCry1Ac toxin protein expression level was performed using Enzyme-Linked Immunosorbent Assay (ELISA). Wild-type 84K poplar was used as the negative control, and no specific toxin protein expression was detected in it. Toxin protein expression was detected in all 16 progenies carrying the *BtCry1Ac* gene, and the expression levels showed a certain degree of inter-group and intra-group variation. Quantitative analysis results showed (Figure 6a) that the toxin protein content of the maternal parent Pb29 was 0.088 pmol/mg, while differences in toxin protein expression existed among hybrid progeny combinations, but the fluctuation range was relatively small. Group C exhibited the highest toxin protein accumulation level (0.108 pmol/mg), significantly higher than group A, which had the lowest expression (0.075 pmol/mg); the toxin protein content of hybrid combination B was 0.106 pmol/mg, also significantly higher than group A. Expression variation existed within each hybrid combination to varying degrees. Group A toxin protein expression levels (Figure 6b) showed significant intra-group variation characteristics, with the highest-expressing individual A4 (0.119 pmol/mg) differing by nearly three times from the lowest-expressing individual A3 (0.040 pmol/mg), with a CV of 37.91%, slightly higher than the overall inter-combination variation level (34.82%); group B expression was the most stable (CV = 22.27%). Although the difference between the highest-expressing individual B6 (0.132 pmol/mg) and the lowest-expressing individual B3 (0.085 pmol/mg) was relatively small, statistical testing indicated that this difference was significant (Figure 6c). Group C had a CV of 36.98%, with significant differences between its extreme expressing individuals. The highest-expressing individual C5 (0.150 pmol/mg) was nearly three times higher than the lowest-expressing individual C3 (0.053 pmol/mg) (Figure 6d). This result confirms that the foreign *BtCry1Ac* gene can be stably expressed in hybrid progeny, and there are large individual differences in the regulation of foreign protein expression within each hybrid combination.

Association analysis between different insertion sites and toxin protein expression levels revealed that the foreign gene insertion site significantly influenced the expression level of the toxin protein. In terms of expression level (Figure 6e), progeny inheriting a single site on chromosome 3 showed the highest average toxin protein content (0.103 pmol/mg), significantly higher by 94.34% than progeny with a single site on chromosome 10 (0.053 pmol/mg). However, compared to the maternal parent Pb29 (0.088 pmol/mg) and progeny inheriting dual sites (0.094 pmol/mg), the differences were not statistically significant. In terms of expression stability, the dual-site group showed high expression variation (CV = 39.87%), with the highest toxin protein content being 3.75 times higher than the lowest, whereas the expression in the chromosome 3 single-site group was relatively stable (CV = 23.99%). Further correlation analysis showed (Figure 6f) that there was no significant correlation between transcript abundance and toxin protein content. Although the transcription levels of the *BtCry1Ac* gene in all hybrid progenies were lower than the maternal parent, progenies such as A2, A4, and B1 exhibited higher toxin protein accumulation than the maternal parent. This phenomenon suggests that important regulatory mechanisms at the post-transcriptional level (such as mRNA stability, translation efficiency, or protein stability) may exist, thereby affecting the final expression level of the foreign toxin protein.

### 2.7. Ploidy Detection in Hybrid Progeny

**Figure 7:**
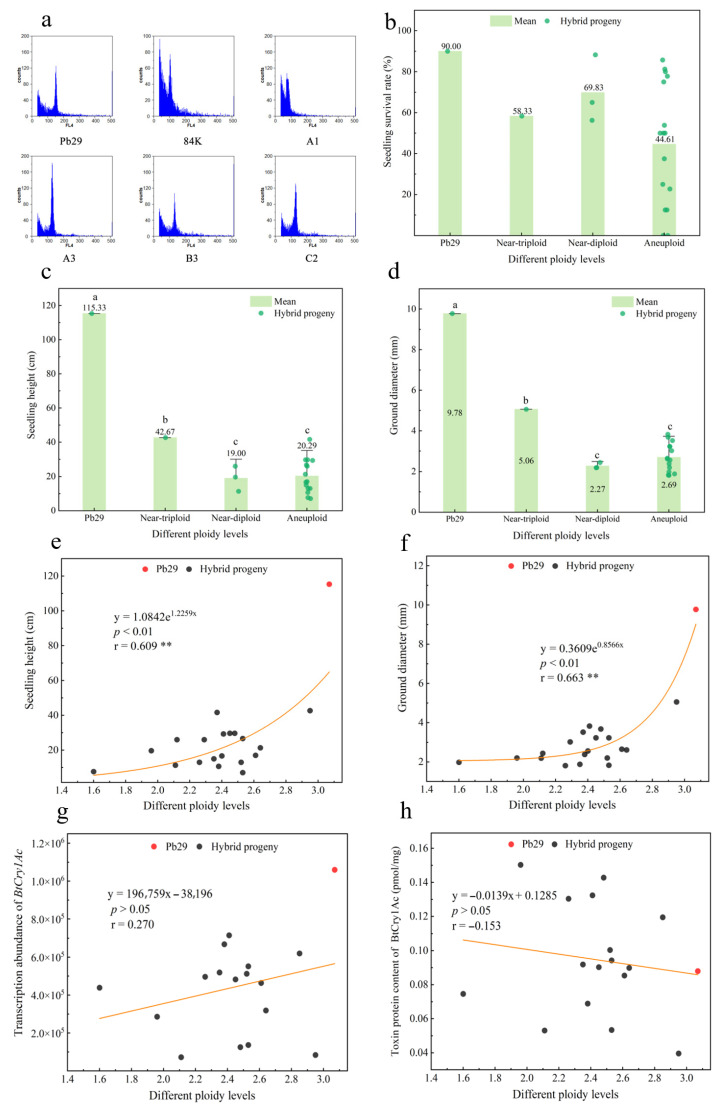
Comparative analysis of survival rates, growth traits, and transcript–toxin correlations in hybrid progeny across ploidy levels. Note: figure (**a**) shows some flow cytometry histograms, corresponding to Pb29, 84K, A1, A3, B3, and C2; figure (**b**) compares the survival rate of hybrid progeny with different ploidy levels; figure (**c**) compares the height of hybrid progeny at different ploidy levels; figure (**d**) compares the ground diameter of hybrid progeny at different ploidy levels; figure (**e**) analyzes the correlation between ploidy and height in hybrid progeny at different ploidy levels; figure (**f**) analyzes the correlation between ploidy and ground diameter in hybrid progeny at different ploidy levels (Pb29 is triploid and 84K is diploid); figure (**g**) shows the correlation analysis of transcript abundance for hybrid progeny at different ploidy levels; figure (**h**) presents the correlation analysis of toxic protein content for hybrid progeny at different ploidy levels.Correlation analysis between the ploidy levels of hybrid progeny and the transcript abundance of the *BtCry1Ac* gene and toxin protein expression levels revealed (Figure 7f,g) no significant correlation in the expression characteristics of the exogenous gene between the known triploid Pb29 and the progeny of different ploidy types (including near-triploid, near-diploid, and aneuploid). This result indicates that, within the hybrid population of this study, genomic ploidy variation was not a key factor affecting the expression level of the exogenous gene. The transcription and translation processes of the exogenous gene may be primarily regulated by inserting site-specific factors or other epigenetic mechanisms, showing a weak association with the overall ploidy state of the plant.

Systematic ploidy analysis was performed on 20 hybrid progenies, the triploid maternal parent Pb29, and the diploid poplar 84K using flow cytometry (Supplementary Figure S1). Using the diploid 84K poplar (n = 2) as a reference standard, the chromosomal ploidy of each sample was quantitatively evaluated using the ploidy calculation formula. The results showed that among the tested hybrid progenies, four were inferred to have nearly euploid chromosomal compositions, while the rest had nearly aneuploid chromosomal compositions. The F-value for 84K was 2.06, and the estimated ploidy F-value for the maternal parent Pb29 was 3.07, which closely matched its known ploidy, validating the reliability of the detection method (Figure 7a). Among the hybrid progenies, A3 had an F-value of 2.95, indicating that its chromosomal composition was close to triploid; C3, C4, and C5 had F-values ranging from 1.90 to 2.20, suggesting that their chromosomal compositions were close to diploid. In contrast, A1 had an F-value of 1.60, while the F-values of the remaining progenies were distributed in the range of 2.20 to 2.90. The variation in these values suggests that these progenies likely exhibit aneuploid characteristics, presumably resulting from unstable chromosome segregation in the triploid maternal parent during hybridization. Based on the above flow cytometry analysis results, the hybrid progenies were categorized into three ploidy types: near-triploid, near-diploid, and aneuploid. An analysis of the survival rates of the maternal parent Pb29 and its hybrid progeny revealed (Figure 7b) that the maternal parent Pb29 exhibited a survival rate of 90.00% during planting, while the survival rates of all hybrid progenies were lower than that of the known triploid control. In terms of growth traits (Figure 7c,d), the maternal parent Pb29 significantly outperformed all hybrid progenies in both seedling height and ground diameter. The near-triploid progeny showed significantly better performance in seedling height and ground diameter growth compared to the near-diploid and aneuploid types within the hybrid progeny. Among the hybrid progenies themselves, the aneuploids exhibited superior growth performance compared to the near-diploids. These results suggest that the near-diploid and near-triploid progenies among the hybrids may not be complete euploids. Correlation analysis results showed (Figure 7e,f) a highly significant positive correlation between plant ploidy and growth traits. As the ploidy level increased, both seedling height and ground diameter showed obvious increasing trends, with correlation coefficients of r = 0.609 (height) and r = 0.663 (ground diameter), respectively. This result reveals the important regulatory role of the ploidy effect on plant growth traits.

### 2.8. Insect Resistance Detection in Progeny Carrying the BtCry1Ac Gene

**Figure 8:**
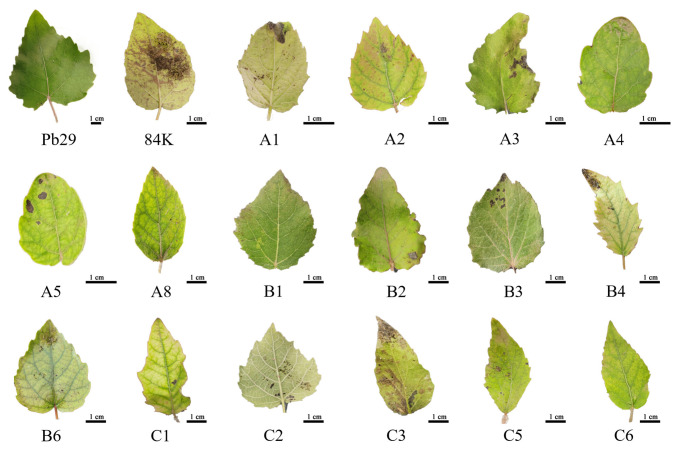
Leaf feeding condition after 2 days of insect bioassay.

**Figure 9:**
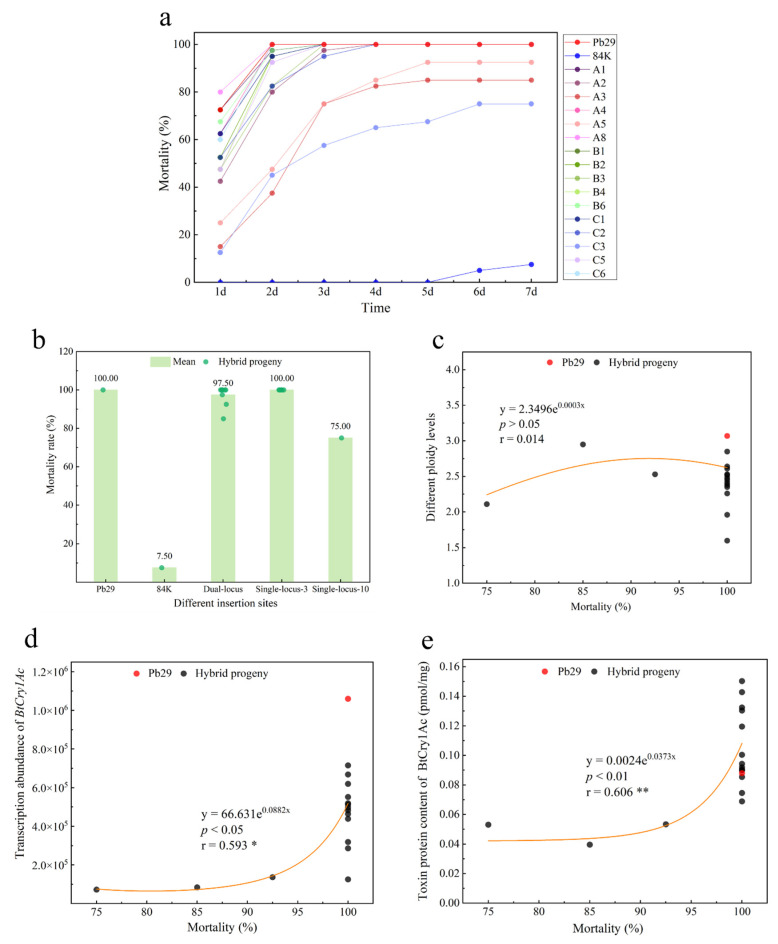
Analysis of insect mortality rates and molecular detection indicators in hybrid progeny. Note: figure (**a**) shows the scatter distribution of insect mortality rates for hybrid progeny; figure (**b**) presents the mortality rates of *Hyphantria cunea* larvae fed on hybrid progeny with different insertion sites; figure (**c**) provides an analysis of the correlation between insect mortality rates of hybrid progeny and ploidy; figure (**d**) offers an analysis of the correlation between insect mortality rates of hybrid progeny and transcript abundance; figure (**e**) illustrates the correlation between insect mortality rates of hybrid progeny and toxin protein content.

An analysis of leaf consumption in feeding trials with various clones revealed (Figure 8 and Figure 9a) that the overall mortality rate of first-instar *Hyphantria cunea* larvae fed on transgenic progeny leaves reached 97.03%, with notable differences in insect resistance efficacy among different clones. The leaves of the non-transgenic control, 84K poplar, exhibited obvious damage symptoms; the leaves were heavily consumed. The maternal parent Pb29 and its progenies A4, A8, B1, and C6 achieved 100% mortality due to their high lethality against *Hyphantria cunea* larvae; thus, their leaves remained relatively intact. In contrast, over half of the larvae survived on A3, A5, and C3, with these progeny clones showing visibly greater leaf damage than other hybrid lines. All tested hybrid progenies exhibited varying degrees of insect resistance; some (such as A4, A8, B1, and C6) showed insect resistance efficacy comparable to Pb29, while A3, A5, and C3, although slightly weaker, were still superior to the non-transgenic control. This result confirmed that the *BtCry1Ac* gene could be stably expressed and could exert insecticidal effects in the hybrid progeny, although there were certain differences in insect resistance among the different progeny clones.

Data from the larval mortality rate trial (Figure 9a) and mortality index (Supplementary Table S4) showed that the maternal parent Pb29 exhibited extremely strong insecticidal activity, with a larval mortality rate of 100.00%, whereas the larval mortality rate of the wild-type 84K poplar was only 7.50%, showing no significant insect resistance effect. Daily monitoring results showed that hybrid progeny clones exhibited larval mortality rates ranging from 12.50% to 80.00% just one day after feeding, significantly higher than the zero mortality rate in the control group. By the second day of the experiment, A4, A8, B1, and C6 had achieved a 100% mortality rate, and the mortality rates of the other clones also increased to 37.50–97.50%. At the end of the experiment (day 7), except for A3, A5, and C3, which still had a small number of surviving larvae, all other progenies achieved complete lethality (Supplementary Table S3). The final larval mortality rates of all hybrid progenies exceeded 75%. Among them, the larval mortality index of B1 (0.18) was comparable to Pb29, while A8 (0.17) even exhibited insecticidal toxicity superior to the maternal parent. The results confirmed that the *BtCry1Ac* gene could be stably inherited and could maintain high insecticidal activity in the hybrid progeny, but there were substantial differences in insecticidal efficiency among different progenies, which might be related to variations in transgene expression levels or the influence of other genetic factors.

A comparative analysis of larval mortality rates among hybrid progeny with different insertion sites revealed (Figure 9b) that progeny inheriting a single site on chromosome 3 exhibited the same 100% mortality rate as Pb29. Progeny carrying dual sites had a mortality rate of 97.50%, while progeny inheriting only a single site on chromosome 10 had the lowest mortality rate of 75.00%. This result indicates that the single insertion site on chromosome 3 possesses more efficient gene expression capability. In contrast, the single insertion site on chromosome 10 may lead to suppressed expression of the exogenous gene due to a position effect. When inherited together with the single site on chromosome 3, it may reduce the overall insect resistance efficacy of the progeny. Further correlation analysis showed (Figure 9c) no significant correlation between ploidy level and mortality rate. However, transcript abundance showed a significant positive correlation with mortality rate (Figure 9d, r = 0.593). Except for A5 (transcript abundance, 1.37 × 10^5^ copies; mortality rate, 92.50%), all progenies with transcript abundance higher than 1.26 × 10^5^ copies achieved 100% mortality. Moreover, toxin protein content showed a highly significant positive correlation with mortality rate (Figure 9e, r = 0.606). A toxin protein content of 0.0689 pmol/mg in the progeny ensured complete lethality. These results confirmed that the insertion site of the exogenous gene determines the insect resistance efficacy of transgenic plants by influencing its expression level, and the accumulated toxin protein content can serve as a reliable indicator for predicting insecticidal activity.

**Figure 10:**
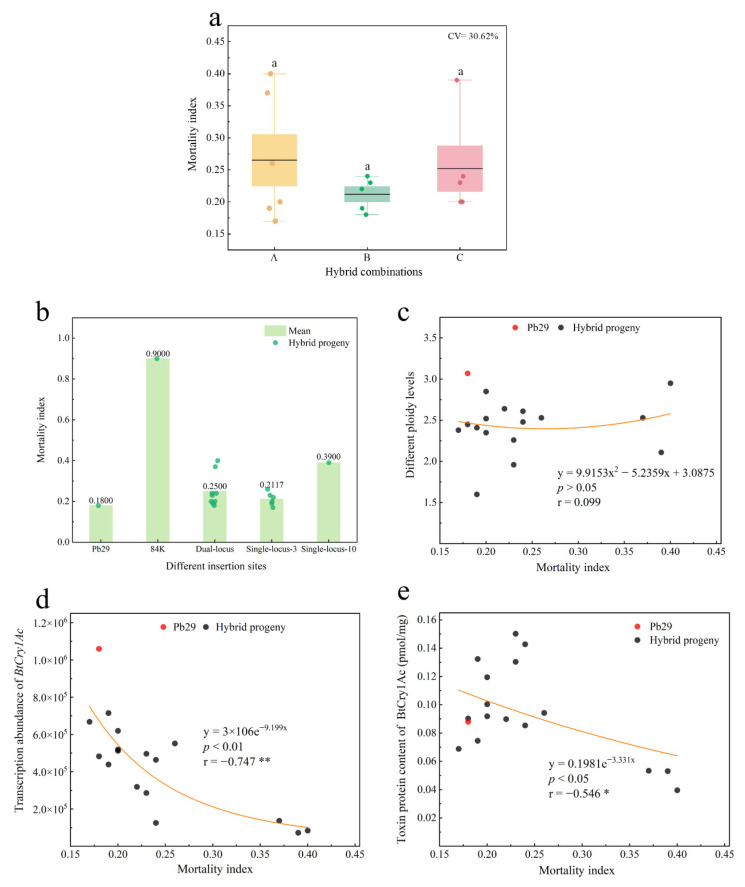
Variation in insect resistance mortality index and its correlation with *Bt* gene expression indicators in hybrid progeny. Note: figure (**a**) shows the mortality index of various hybrid progeny combinations; figure (**b**) presents an analysis of genetic loci and the mortality index of hybrid progeny; figure (**c**) provides an analysis of the correlation between the mortality index and ploidy; figure (**d**) examines the correlation between the mortality index and transcript abundance in hybrid progeny; figure (**e**) analyzes the correlation between the mortality index and toxin protein content in hybrid progeny.

A comparative analysis of the insecticidal toxicity among the three hybrid combinations revealed (Figure 10a) that the differences in mortality index between the combinations were not statistically significant, with Group B exhibiting the strongest average insecticidal toxicity. Group A showed large variation in insecticidal toxicity (CV = 37.03%), containing both the progeny with the best resistance (A8) and the worst resistance (A3) among all progenies; the resistance within Group C (CV = 31.42%) was also relatively discrete. In contrast, Group B (CV = 12.20%) exhibited the most stable resistance, indicating that the paternal genetic background influences the stability of insecticidal toxicity in the progeny. Further analysis showed (Figure 10b) that the insertion site of the exogenous gene plays an important regulatory role in insecticidal toxicity. The average resistance of progeny with a single site on chromosome 3 was similar to that of the maternal parent Pb29; progeny with a single site on chromosome 10 performed the worst, and progenies with dual sites were intermediate. Furthermore, the stability of the single site on chromosome 3 (CV = 15.06%) was higher than that of the dual sites (CV = 31.94%). Correlation analysis indicated that insecticidal toxicity showed no significant correlation with ploidy (Figure 10c, r = 0.099) but exhibited a highly significant negative correlation with transcript abundance (Figure 10d, r = −0.747) and a significant negative correlation with toxin protein content (Figure 10e, r = −0.546).

## 3. Discussion

This study used the highly abortive transgenic poplar triploid variety Pb29 as the maternal parent for controlled pollination hybridization. Due to reduced gamete fertility caused by abnormal chromosome segregation during meiosis [22], this material exhibits significant reproductive barriers, including poor catkin development (70–90% shedding before maturity) and high rates of embryo abortion after natural pollination [23]. This shares biological homology with the mechanisms underlying the widespread reproductive barriers and seedless traits in triploid plants [24]. Embryo rescue technology is used to obtain seedlings from hybrid embryos of different plant species. It is a technique that converts hybrid embryos (intra- and interspecific hybrids that are nonviable under normal conditions) into living plants with the aid of an in vitro plant growth environment [25]. To successfully obtain hybrid progenies from poplars of different ploidies, the experiment applied embryo rescue methods to promote the development of immature embryos. A total of 104 immature embryos were obtained at the tissue culture stage from the three hybrid combinations. Comparative studies of the three paternal materials found that although 84K poplar exhibited high pollen viability, the germination rate of its hybrid immature embryos did not reach a significant difference level compared to the other paternal parents. This aligns with previous research concluding that the maternal genotype plays a dominant role in embryo rescue efficiency [26]. The pollen of *Populus × euramericana* Male No. 1 showed unique patterns of viability changes under 4 °C storage conditions. Preliminary analysis suggests that this may be related to its pollen grain morphological characteristics and post-maturation physiological properties. The hybrid combination using 84K poplar as the paternal parent not only yielded more progenies, but its progeny also showed significant advantages in seedling growth and leaf shape variation; in particular, lines A5, A6, and A8 exhibited distinct differences in leaf shape characteristics compared to the maternal parent, as well as rich polymorphism among themselves. These results suggest that 84K poplar may have superior reproductive compatibility with the maternal parent Pb29.

Ploidy analysis via flow cytometry showed that the hybrid progeny population exhibited variations in chromosome number, fully confirming that abnormal chromosome segregation during meiosis in the triploid maternal parent can lead to aneuploid formation [22]. Kojima et al. proposed that the dosage compensation effect of homologous genes in plant genomes is more pronounced in aneuploids, yet aneuploid plants still exhibit lower survival rates [27]. The experiment selected developing seeds for embryo rescue, but a large number of embryos died during the tissue culture stage, and the field survival rate of transplanted surviving progeny was lower than that of the maternal parent Pb29. This phenomenon may be directly related to the ploidy abnormalities in the progeny. Further analysis of the results found that the hybrid progenies were significantly lower than the maternal parent Pb29 in terms of seedling height, ground diameter, and survival rate. In particular, the survival rates of the four near-euploid progeny lines (including near-triploid A3 and near-diploids C2, C4, C5) were all lower than that of the known triploid Pb29. Among them, the growth indices of near-triploid A3, while lower than Pb29, were superior to those of the near-diploids. Wang et al. observed that in triploid Populus ‘Yinzhong’ hybrids, most aneuploid progenies exhibited inferior growth traits (e.g., plant height and ground diameter) compared to both parental lines and euploid progeny [28]. Based on these results, it is hypothesized that even though the ploidy of these progenies is close to euploid, there may still be monosomy or trisomy of 1–2 chromosomes. This hypothesis requires further verification using more precise methods. Furthermore, aneuploid progeny exhibited obvious phenotypic variations. For instance, line C3 simultaneously displayed dual phenotypic traits of abnormal leaf shape and defective development of basal lateral branches. This may be due to the imbalanced expression of dosage-sensitive genes caused by chromosome number variation, thereby affecting organ developmental patterns. Such morphological variations are not only regulated by the paternal genetic background, as demonstrated by Luo et al. regarding the dominant effect of the paternal parent on fruit traits in a jujube hybrid system [29], but are also closely related to gene dosage effects induced by chromosomal aneuploidy [30] and disruptions in developmental regulatory networks [31]. For example, research by Bai et al. on rapeseed aneuploids revealed that changes in the copy number of chromosome A09 could lead to deviations in homologous gene expression and trigger phenotypic differentiation in flowering time [32]; Mei et al. confirmed through tandem duplication of the DHN gene cluster that increased gene dosage can enhance drought survival rates in *Arabidopsis* seedlings by 37.5% [33]. Given that plant morphogenesis is governed by complex transcriptional regulatory networks [34], aneuploidy may disrupt the precise regulation of meristem activity and organ boundary establishment by impairing the dosage balance of key regulatory factors, such as the SUP gene family [35] and the DJ-1 gene family [36].

According to Mendel’s law of independent assortment, two exogenous genes located on non-homologous chromosomes are expected to produce progeny with dual sites, single sites, and no insertion at a ratio of 25% each [37]. However, in this study, the inheritance frequency of the *BtCry1Ac* gene (80%) significantly deviated from the theoretical value. This abnormal genetic phenomenon may result from the aberrant meiotic characteristics of the triploid maternal parent Pb29, or alternatively, from the *Bt* gene-containing gametes possessing stronger selective advantages and biological activity, thereby producing gamete types that deviate from Mendelian inheritance laws. Further validation should be conducted by expanding the hybrid offspring population size. Systematic analysis of T-DNA integration patterns is a key step in assessing the potential risks and environmental impacts of genetically modified organisms [38]. Using exogenous gene insertion site-specific primers, the integration pattern of T-DNA in the hybrid progeny population was analyzed. The results showed that nine progeny lines were the same as the maternal parent Pb29, carrying both insertion sites; six lines retained only the insertion site on chromosome 3, while line C3 specifically inherited the insertion site on chromosome 10. Chen et al. suggested that the presence of multiple copies of exogenous genes within transgenic crops can interfere with each other, thereby affecting gene expression [39]. In this experiment, progeny inheriting only the single site on chromosome 3 had significantly higher average transcript abundance and toxin protein content compared to progeny with the single site on chromosome 10, but the difference compared to dual-site progeny did not reach statistical significance, while dual-site progenies were significantly higher than those with the single site on chromosome 10; progeny with the single site on chromosome 3 had superior average mortality rates and mortality indices compared to dual-site progeny, while dual-site progenies were superior to those with the single site on chromosome 10. This result suggests that the foreign gene may be subject to differential epigenetic regulation at different insertion sites, and the insertion site on chromosome 10 may have a stronger gene silencing effect. Moreover, in the transgenic hybrid progeny of poplar from this experiment, potential gene silencing mechanisms may include not only co-suppression but also position effects [40]. Subsequent studies should investigate gene silencing effects and DNA methylation patterns to systematically elucidate the molecular mechanisms underlying reduced *BtCry1Ac* expression in progeny with the single insertion site on chromosome 10. Simultaneously, gene expression might also be influenced by aneuploidy. The experimental results showed that dual-site progeny A3 had the lowest insect mortality rate and insecticidal toxicity among all progenies, with a ploidy of 2.95, close to triploid; progeny carrying only the site on chromosome 3 achieved 100% mortality by the second day of the insect bioassay and had insecticidal toxicity similar to Pb29, while the ploidy of this group ranged from 2.26 to 2.64; C3 carrying the site on chromosome 10 had aneuploid characteristics (F = 2.11) and exhibited lower mortality rates and insecticidal toxicity than progeny with the site on chromosome 3 during the 7-day insect bioassay. Furthermore, having only one clone with a single copy on chromosome 10 may not comprehensively reflect the impact of the chromosome 10 insertion site on gene expression. In summary, the expression of the exogenous gene may be influenced by multiple factors, including copy number, insertion position, and individual ploidy. Expanding the hybrid population and conducting in-depth research is necessary.

Previous studies have shown that the BtCry1Ac protein must reach a certain concentration to cause damage to the midgut structure and death of midgut epithelial cells in lepidopteran insects [41]. Among the various detection indicators in this study, toxin protein content showed a highly significant correlation with insect mortality rate. A toxin protein content of 0.0689 pmol/mg was sufficient to achieve complete lethality. This explains why some dual-site progenies, although having lower toxin protein content than Pb29, still exhibited 100% mortality like Pb29. However, no significant correlation was observed between transcript abundance and toxin protein content. This phenomenon suggests that more complex regulatory mechanisms at the post-transcriptional level (such as mRNA stability, translation efficiency, and protein modification) may influence the final insect-resistant phenotype.

This study used a female transgenic triploid poplar as the maternal parent and successfully cultivated a series of aneuploid hybrid progenies through hybrid combinations with three different paternal parents, obtaining valuable experimental materials for poplar aneuploid genetic research. Preliminary research was conducted on the growth traits and morphological observations, exogenous gene integration and expression, ploidy characteristics, and hybrid insect resistance of the progeny. Preliminary results showed that the A3 hybrid progeny exhibited optimal growth performance, while A8 and B6 maintained insect resistance comparable to the maternal parent Pb29. In subsequent studies, parental lines and progeny materials will be vegetatively propagated and transplanted to field conditions for phenotypic observation. Upon trait stabilization, comprehensive evaluations including *BtCry1A* gene expression analysis, *BtCry1A* toxin protein quantification, insect resistance assessment, and growth parameter measurements will be conducted to select elite insect-resistant aneuploid *Populus tomentosa* germplasm. Concurrently, phenotype-directed gene discovery will be pursued via cloning and functional validation across aneuploid lines. To further verify the reliability of the research findings, subsequent work will expand the range of parental selection to obtain materials with different copy numbers and various aneuploid types. Advanced techniques such as karyotype analysis, ATAC-seq, and BS-seq will be used to precisely determine the ploidy of hybrid progeny and deeply analyze the epigenetic characteristics of the exogenous gene insertion sites. Multi-generational tracking of insect resistance will also be conducted to assess trait stability.

## 4. Materials and Methods

### 4.1. Plant and Insect Materials

This study used the highly resistant transgenic *BtCry1Ac* poplar 741 line Pb29 [3] as the maternal parent for hybridization. Pb29 is a triploid insect-resistant variety that has undergone years of insect resistance and environmental safety testing, has obtained a safety certificate, and is commercially cultivated, exhibiting high resistance to lepidopteran pests [42,43]. *Populus* 84K (*Populus alba × Populus glandulosa*), *Populus leucopyramidalis,* and *Populus × euramericana* Male No. 1 (*Populus × euramericana* NO. 1) were used as paternal parents (Supplementary Table S1). Hybrid parent materials were provided by the Tree Breeding Laboratory, College of Forestry, Hebei Agricultural University. Branches were collected in March 2023. Larvae of *Hyphantria cunea* used in feeding experiments were collected from the State-owned Forest Farm in Luannan County, Tangshan City.

### 4.2. Paternal Pollen Viability and Hybridization Methods

Paternal branches with flower buds were collected, cut into 50–80 cm segments, and placed in buckets with clean water for hydroponics. The culture water was changed every 48 h. When mature pollen appeared at the lower end of the male catkins, the catkins were gently tapped to release pollen onto a Petri dish below. The dish was sealed with plastic wrap and stored in desiccators at room temperature and at 4 °C. Every 72 h, a brush was used to evenly spread an appropriate amount of pollen onto the surface of the pollen viability assay medium (containing 10% sucrose, 0.01% boric acid, 1% agar, pH 6.0 ± 0.3). After dark incubation at a constant 25 °C for 12 h, pollen germination was observed under an optical microscope, and the pollen germination rate was calculated. Subsequently, a brush dipped in pollen was used to lightly touch the stigma for pollination. Bagging was applied before and after pollination to prevent pollen contamination. After fruit entered the maturation stage, bags were replaced with mesh bags to ensure ventilation.

### 4.3. Embryo Rescue and Hybrid Progeny Propagation

Catkins 7–9 days after pollination were placed in a laminar flow hood, immersed in 20% NaClO solution for 1 min, rinsed three times with sterile water, and then immersed in 75% ethanol for 1 min. Capsules were separated from the catkins, pericarps were peeled off, and well-developed immature embryos were selected and inoculated onto embryo rescue medium (1/2MS + 0.1 mg/L 6-BA + 0.05 mg/L IAA + 30 g/L sucrose + 6.5 g/L agar). Twenty embryos were inoculated per Petri dish and cultured in a tissue culture room at 25 °C with a photoperiod set to 16 h light/8 h dark. After embryo development, the germination rate was calculated. The materials were then transferred to differentiation medium (MS + 0.2 mg/L 6-BA + 0.1 mg/L IBA + 30 g/L sucrose + 6.5 g/L agar) for propagation culture. The medium was replaced every 20 days to obtain robust adventitious buds. After sufficient adventitious buds were obtained through subculturing, the materials were standardized by pruning to ensure basic consistency in growth traits such as seedling height and leaf number. Subsequently, they were inoculated onto rooting medium (1/2MS + 0.5 mg/L IBA + 15 g/L sucrose + 6.5 g/L agar) for root induction.

### 4.4. Measurement of Seedling Survival Rate and Growth Traits in Hybrid Progeny

Two months after transplanting tissue-cultured seedlings of the maternal parent Pb29 (n = 3) and hybrid progeny to the field, phenotypic traits of seedling height and ground diameter were measured using a tape measure and an electronic caliper. Each indicator was measured three times. A digital camera was used to record morphological characteristics such as the leaf shape and plant type of the hybrid progeny. Three months after transplanting, the field survival rate of clonal hybrid progeny seedlings was recorded.

### 4.5. PCR Detection of the BtCry1Ac Gene

DNA was extracted from Pb29 and hybrid progeny clones using the CTAB method. PCR amplification detection was performed using specific primers Bt-F/Bt-R (Bt-F: TGAATGGACATGCATGGGATGG, Bt-R: CCACCTTTGTCCAAACACTGAA) and Kan-F/Kan-R (Kan-F: ATGACTGGGCACAACAGACAATC, Kan-R: TATGTCCTGATAGCGGTCCGC). The PCR reaction program was as follows: initial denaturation at 98 °C for 3 min; 30 cycles of denaturation at 98 °C for 10 s, annealing at 50 °C for 10 s, extension at 72 °C for 15 s; final extension at 72 °C for 5 min.

### 4.6. Detection of Foreign Gene Insertion Sites in Hybrid Progeny

There are two T-DNA insertion sites in the Pb29 genome, located at positions 9,283,905–9,283,937 bp on chromosome 3 and 10,868,777–10,868,803 bp on chromosome 10, respectively. Reported specific primer combinations 3 (Chr3u-F1/131#S2F), 4 (Chr3d-R2/131#S5F), 6 (Chr10u-F2/131#S5F), and 7 (Chr10d-R2/131#S2F) (Supplementary Table S2) were used to identify the two foreign gene insertion sites and analyze T-DNA insertion orientation in hybrid progeny clones [21]. PCR reaction conditions were as follows: initial denaturation at 98 °C for 3 min; 32 cycles of denaturation at 98 °C for 10 s, annealing at 60 °C for 10 s, extension at 72 °C for 25 s; final extension at 72 °C for 5 min.

### 4.7. qPCR Detection of the BtCry1Ac Gene

Total RNA was extracted from the 3rd to 5th apical leaves of Pb29 and hybrid progeny containing the *BtCry1Ac* gene, and reverse transcribed into cDNA. Specific quantitative real-time PCR (qPCR) primers Bt-F/Bt-R (qBt-F: GAATTTTTGGTCCCTCTCAAT, qBt-R: AGGATCTGCTTCCCACTCTCT) were designed. Using Tian Gen 2×Fast Rea-l qPCR Pre Mix (SYBR Green) reagent (Beijing, China), the relative expression level of the *BtCry1Ac* gene was detected using an Agilent Mx3005P real-time fluorescence quantitative PCR system (Santa Clara, CA, USA). The qPCR reaction program was as follows: pre-denaturation at 95 °C for 2 min; 40 cycles of denaturation at 95 °C for 5 s, extension at 60 °C for 30 s. All experiments were independently repeated three times.

### 4.8. ELISA Detection of Bt Toxin Protein in Hybrid Progeny

This study used an ELISA kit (F50074-A) purchased from Fan Ke Wei (Shanghai, China) to quantitatively analyze the total protein content and BtCry1Ac toxin protein content in each experimental line. Pb29 was used as the positive control and 84K poplar as the negative control. To ensure the reliability of the experimental results, each sample was tested in three independent replicates. Absorbance was measured using a microplate reader at a detection wavelength of 450 nm, with three replicates for all samples. Data analysis used the standard curve method. By establishing a linear relationship between standard protein concentration and absorbance, the content of the BtCry1Ac protein in the samples was calculated.

### 4.9. Ploidy Verification of Hybrid Progeny

Hybrid progenies were entrusted to Jindi Future Biotechnology Co., Ltd. (Beijing, China) for ploidy detection. Samples consisted of young leaves approximately 2 cm^2^ in size. Known-ploidy diploid 84K poplar and triploid transgenic poplar 741 Pb29 were used as control samples. Data analysis was performed using FlowJo 10 software to calculate the relative fluorescence intensity (RFI) of each tested sample. Ploidy was calculated using the following formula—ploidy coefficient (F) = RFI of test sample/RFI of reference sample—from which the ploidy value of the test sample was estimated. The ploidy formula [44] is as follows:

(1) $$F\;=\;\frac{I_{s}}{I_{ck}}\times n$$

where *F* represents the estimated ploidy value of the sample, $I_{s}$ represents the relative fluorescence intensity of the test sample, $I_{ck}$ represents the relative fluorescence intensity of the reference sample, and *n* represents the ploidy of the reference sample.

### 4.10. Insect-Feeding Assay of Hybrid Progeny

The 3rd to 5th apical young leaves from each progeny and parent plant were collected as experimental material. The petioles were inserted into pre-punctured 1.5 mL centrifuge tubes, and deionized water was replenished every 12 h to maintain leaf freshness. The prepared leaf samples were placed in dry, breathable plastic Petri dishes, and each leaf was evenly inoculated with 20 newly hatched first-instar larvae. During the experiment, fresh leaves from each clone were replaced every 48 h, and the number of dead larvae was recorded daily at fixed times. The experiment was terminated when larval mortality stabilized and showed no significant change (usually lasting 7–10 days), and the mortality rate and mortality index were calculated.

(2) $$\mathrm{Mortality}\;\mathrm{rate}=\frac{Number\;of\;dead\;insects}{Total\;inoculated\;insects}\times 100\%$$

(3) $$\mathrm{Mortality}\;\mathrm{index}=\frac{\left(\sum Days\times Daily\;mortality\;count\right)}{\left(Cumulative\;mortality\times Total\;duration\;of\;experiment\right)}$$

### 4.11. Data Processing and Software

Data preprocessing was organized and summarized using Microsoft Excel 2019 software. Statistical analysis was performed using SPSS 27.0 software (IBM, New York, NY, USA). Multi-group data comparison used one-way analysis of variance (one-way ANOVA), with the significance level set at α = 0.05. Graphs were plotted using Origin 2021 software (Origin Lab, Northampton, MA, USA).

## 5. Conclusions

Using the transgenic triploid poplar 741 variety Pb29 carrying the *BtCry1Ac* gene as the maternal parent, crosses were performed with three different paternal parents, respectively. A total of 20 transplanted and surviving hybrid progenies were successfully obtained using embryo rescue technology. The results showed that when 84K poplar was used as the paternal parent, it exhibited the best gamete compatibility with the maternal parent, and its hybrid progeny had significant advantages in growth traits. Sixteen progenies carried the maternal *BtCry1Ac* gene, and this gene showed linked inheritance characteristics with the selectable marker gene *Kan*. Preliminary determination indicated that all hybrid progenies were aneuploid. These progenies exhibited rich polymorphism in phenotypic characteristics, including variations in seedling height, ground diameter, and leaf shape, but their growth indices were generally lower than those of the maternal parent Pb29. The exogenous gene showed significant expression differences in progenies with different insertion sites (chromosome 3 and chromosome 10). This difference directly led to variations in toxin protein content and insect resistance performance among the progenies. The expression level of the exogenous gene may be co-regulated by multiple factors, including gene copy number, insertion site, and individual ploidy. The aneuploid hybrid progenies created in this study provide valuable research and breeding materials for the selection of new insect-resistant poplar varieties. Subsequent work will involve systematic insect resistance testing and growth trait monitoring to screen for new germplasm with superior characteristics.

## Data Availability

Data sharing is not applicable to this article as no datasets were generated or analyzed during the current study.

## Supplementary Materials

The following supporting information can be downloaded at https://www.mdpi.com/article/10.3390/plants14162563/s1, Table S1. Plant Materials. Table S2. Primer Information. Table S3. Statistics of Insect Bioassay Results. Table S4. Measurement Information of Various Indicators for Hybrid Progeny. Figure S1. Flow Cytometry Results of Pb29, 84K and Hybrid Progeny.
