# Supplementary material for: Analysis of Insect Resistance and Ploidy in Hybrid Progeny of Transgenic BtCry1Ac Triploid Poplar 741
Source: Plants (Basel). 2025 Aug 18;14(16):2563. doi: 10.3390/plants14162563 (PMC12389618; doi:10.3390/plants14162563)

Supplementary Table S1. Plant Materials

Variety/Clone	Female parent	Male parent
Populus 741	*Populus alba* × (*Populus davidiana* + *Populus simonii*)	*Populus tomentosa*
Populus 84K	*Populus alba*	*Populus glandulosa*
Populus leucopyramidalis	*Populus hopeiensis* ×*Populus tomentosa*	*Populus tomentosa*×*Populus alba var. pyramidalis*
Populus × *euramericana* Male No.1	*Populus deltoides*	*Populus nigra*

Supplementary Table S2. Primer Information

Primer name	Sequences (5' to 3')
Chr3u-F1	AGAGTACGCCCTTTGATTATTTGCT
Chr3d-R2	GCCTGACATTGCGGTGACATTCTGC
Chr10u-F2	CGACGAGATGCCTCCACCATTCTGA
Chr10d-R2	TCTTCTATGGTTGCTCCTGCTTTGT
131#S2F	ATTTGGGTGATGGTTCACGTAGTGG
131#S5F	TAGTGACCTTAGGCGACTTTTGAACG

Supplementary Table S3. Statistics of Insect Bioassay Results

Strain	Mortality rate（%）						
	1 d	2 d	3 d	4 d	5 d	6 d	7 d
PB29	72.50	100.00	-	-	-	-	-
84K	-	-	-	-	-	5.00	7.50
A1	72.50	97.50	100.00	-	-	-	-
A2	42.50	80.00	97.50	100.00	-	-	-
A3	15.00	37.50	75.00	82.50	85.00	85.00	85.00
A4	62.50	100.00	-	-	-	-	-
A5	25.00	47.50	75.00	85.00	92.50	92.50	92.50
A8	80.00	100.00	-	-	-	-	-
B1	72.50	100.00	-	-	-	-	-
B2	52.50	95.00	100.00	-	-	-	-
B3	47.50	82.50	100.00	-	-	-	-
B4	47.50	95.00	100.00	-	-	-	-
B6	67.50	97.50	100.00	-	-	-	-
C1	62.50	95.00	100.00	-	-	-	-
C2	52.50	82.50	95.00	100.00	-	-	-
C3	12.50	45.00	57.50	65.00	67.50	75.00	75.00
C5	47.50	92.50	100.00	-	-	-	-
C6	60.00	100.00	-	-	-	-	-

Supplementary Table S4. Measurement Information of Various Indicators for Hybrid Progeny

Strain	Insertion site	Relative fluorescence intensity	F-value (Fluorescence intensity ratio)	Transplant survival rate	*BtCry1Ac* transcript abundance	Toxin content (pmol/mg)	Mortality	Mortality index
84K	-	102.91	2.06	100.00%	-	-	7.50	0.90
PB29	Dual-locus	151.86	3.07	90.00%	1.06×10^{6}	0.08800	100.00	0.18
A1	Dual-locus	82.50	1.60	12.50%	4.39×10^{5}	0.07460	100.00	0.19
A2	Single-locus-3	130.10	2.53	75.00%	5.52×10^{5}	0.09430	100.00	0.26
A3	Dual-locus	151.82	2.95	58.33%	8.43×10^{4}	0.03960	85.00	0.40
A4	Dual-locus	146.85	2.85	0.00%	6.20×10^{5}	0.11950	100.00	0.20
A5	Dual-locus	130.35	2.53	12.50%	1.37×10^{5}	0.05340	92.50	0.37
A6	-	122.17	2.37	53.85%	-	-	-	-
A7	-	117.77	2.29	50.00%	-	-	-	-
A8	Single-locus-3	122.70	2.38	25.00%	6.69×10^{5}	0.06890	100.00	0.17
B1	Dual-locus	126.28	2.45	85.71%	4.83×10^{5}	0.09030	100.00	0.18
B2	Single-locus-3	136.09	2.64	37.50%	3.19×10^{5}	0.08990	100.00	0.22
B3	Dual-locus	134.13	2.61	80.00%	4.64×10^{5}	0.08540	100.00	0.24
B4	Single-locus-3	116.21	2.26	0.00%	4.97×10^{5}	0.13040	100.00	0.23
B5	-	123.56	2.40	22.73%	-	-	-	-
B6	Single-locus-3	123.96	2.41	50.00%	7.15×10^{5}	0.13240	100.00	0.19
C1	Dual-locus	120.81	2.35	81.25%	5.20×10^{5}	0.09190	100.00	0.20
C2	Dual-locus	127.43	2.48	77.78%	1.26×10^{5}	0.14280	100.00	0.24
C3	Single-locus-10	108.50	2.11	88.24%	7.27×10^{4}	0.05310	75.00	0.39
C4	-	109.11	2.12	56.25%	-	-	-	-
C5	Dual-locus	100.70	1.96	65.00%	2.86×10^{5}	0.15030	100.00	0.23
C6	Single-locus-3	129.81	2.52	50.00%	5.13×10^{5}	0.10040	100.00	0.20

Supplementary Figure S1. Flow Cytometry Results of Pb29, 84K and Hybrid Progeny

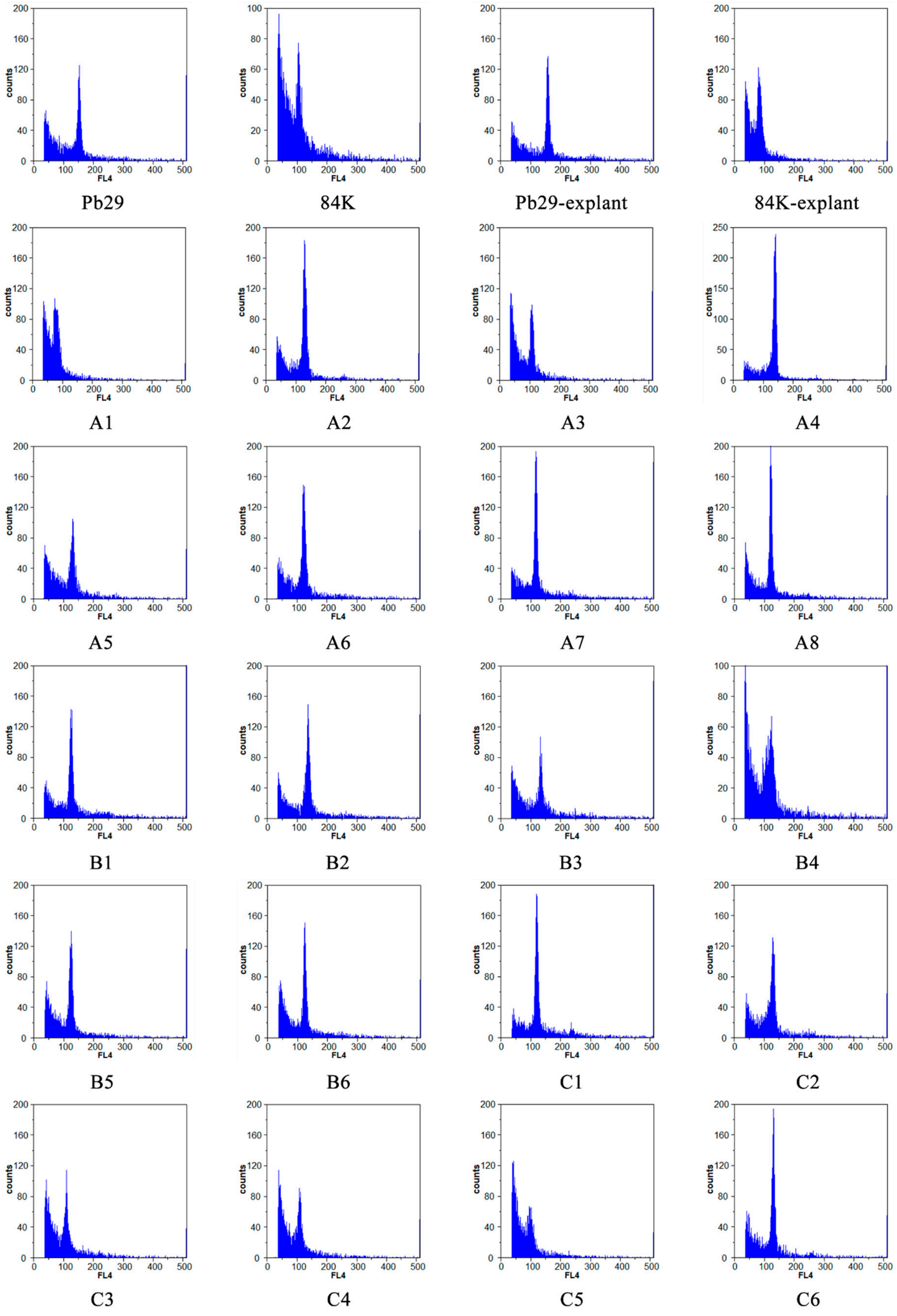

Supplement: Supplementary file 1 [file plants-14-02563-s001.zip › plants-3780053-supplementary.pdf]
